# The Effect of Different Agrotechnical Treatments on the Establishment of *Miscanthus* Hybrids in Soil Contaminated with Trace Metals

**DOI:** 10.3390/plants12010098

**Published:** 2022-12-25

**Authors:** Jacek Krzyżak, Szymon Rusinowski, Krzysztof Sitko, Alicja Szada-Borzyszkowska, Radosław Stec, Elaine Jensen, John Clifton-Brown, Andreas Kiesel, Eva Lewin, Paulina Janota, Marta Pogrzeba

**Affiliations:** 1 Institute for Ecology of Industrial Areas, 6 Kossutha Street, 40-844 Katowice, Poland; 2 Plant Ecophysiology Team, University of Silesia in Katowice, 28 Jagiellońska Street, 40-032 Katowice, Poland; 3 Institute of Biological, Environmental and Rural Sciences, Aberystwyth University, Plas Gogerddan, Aberystwyth SY23 3EB, UK; 4 Biobased Resources in the Bioeconomy (340b), Institute of Crop Science, University of Hohenheim, 70599 Stuttgart, Germany

**Keywords:** biochar, plastic mulch, cadmium, plantation establishment

## Abstract

Climate change and man-made pollution can have a negative impact on the establishment of *Miscanthus* plants in the field. This is particularly important because biomass can be produced on marginal land without conflicting with food crops. The establishment success depends on the hybrid chosen, the cultivation method, the climatic conditions, and the concentration of pollutants in the soil. There are several ways to increase the survival rate of the plants during the first growing season and after the first winter. One of them is the application of biochar and photodegradable plastic mulch, which can provide a solution for soils polluted with trace elements (TMEs). The aim of this study was to investigate the application of plastic mulch and biochar separately and in combination at the planting stage for two *Miscanthus* hybrids planted by the rhizome method (TV1) and seedling plugs (GNT43) on soils contaminated with trace metal elements (Pb, Cd, Zn). TV1 seems unsuitable for TME-contaminated field cultivation, as the survival rate was <60% in most treatments studied. The selected treatments did not increase the survival rate. Furthermore, the application of plastic mulch in combination with biochar resulted in a significant reduction of this parameter, regardless of the hybrid studied. The applied agrotechnology did not influence the TME accumulation in the aboveground plant parts in TV1, while Pb and Cd in GNT43 showed significantly higher values in all treatments. Contrary to expectations, biochar and plastic mulch applied separately and together neither increased survival nor reduced the accumulation of toxic TMEs during establishment on soil contaminated with TMEs and after the first growing season.

## 1. Introduction

Climate change is currently one of the greatest threats, especially with regard to perennial plant establishment [1,2]. *Miscanthus* spp. is classified as an energy crop and is a suitable candidate for cultivation on marginal lands where, despite climate change, other problems occur, such as the presence of pollutants, poor soil quality, and waterlogging, which make food production impossible or economically unviable [3,4,5]. This perennial grass, native to Asia, has low input requirements and is responsible for CO_2_ sequestration and storage in below ground organs, which in turn could be valuable for nutrient-depleted soil, especially due to the translocation of nutrients into the rhizomes after each growing season [6,7,8]. In terms of phytoremediation, there are reports indicating the phytoextraction potential of TMEs by this plant [9,10,11]. However, field trials on such contaminated soils have shown that phytoextraction is insufficient for mature plantations and crops should rather be used for the phytostabilisation process due to the low concentration of TMEs in the aboveground biomass [12,13,14]. Improper establishment alongside susceptible varieties can lead to severe economic losses when the plantation regrows in subsequent years. This risk is particularly high for several *Miscanthus* hybrids [15]. Xue et al. [16] investigated the strengths and weaknesses of different propagation methods for *Miscanthus*, such as rhizome planting, stem-derived plants, direct seed sowing, and micropropagation. Of the propagation methods studied then, the rhizome-derived plant technique and direct seeding were the most important, as rhizome-derived plants have the highest survival success (>85%) and overwintering rate. In contrast, seed propagation has the highest multiplication factor and is the cheapest method among those studied. The serious limitations are the low multiplication rate in the first method, and the sensitivity to water shortage, spring frosts, and low germination rate in the field, respectively. Clifton-Brown et al. [17] proposed an intermediate solution between direct sowing and micropropagation, where the seed is pre-cultivated in the greenhouse and transferred to the field when the seedlings have a greater chance of successfully adapting to field conditions.

Water availability and temperature are the most common factors affecting plant establishment, regardless of species, varieties, and annual or perennial type [1,15,18,19]. Apart from these most common abiotic stress factors, there may be other stress factors in growing energy crops on marginal soils, such as pollutants, i.e., Trace Metal Elements (TMEs), Polycyclic Aromatic Hydrocarbons (PAH) [9,10,20], or nutrient deficiencies [12,13], which may cause a cross-reaction in the plants.

One technique to overcome survival problems due to water scarcity and spring frost during the establishment of *Miscanthus* plants is to cover the field after planting with photodegradable mulch films made of transparent polymer [17]. O’Loughlin et al. [14] concluded that the application of plastic mulch films accelerates the establishment and growth rates of newly seeded rhizome-based *Miscanthus* plants and shortens the time to reach mature biomass yields. Further, Ashman et al. [21] reported in direct seeding of *Miscanthus* that despite there being no increase in germination, overwintering plant failure was reduced.

Trace metal elements are pollutants that are persistent in the environment and, unlike organic pollutants, cannot be biodegraded. Rusinowski et al. [22,23] reported for *Miscanthus* × *giganteus* and various seed-based hybrids that these plants are metal excluders. Furthermore, the biomass yield when grown on good quality soil but contaminated with TMEs is comparable to uncontaminated soil. Nevertheless, some reports indicate a significant stress response, including yield reduction in the initial phase (the first two growing seasons) after transplanting on contaminated soils [24,25]. In particular, the plants accumulate more trace metals in the aboveground organs during the first and second growing seasons than in the following seasons [26].

Aided phytostabilisation can be a solution to reduce the mobility of trace elements (Pb, Cd, Zn) in the soil. Various reports consider different organic [27,28] and mineral additives [29,30,31] with and without plants to reduce the mobility of these elements in the soil. One of the most studied is biochar of different origins [32,33,34]. The role of biochar in aided phytostabilisation is mostly associated with adsorption. The mechanism of biochar adsorption includes surface complexation, hydrogen binding, electrostatic attraction, acid-base interaction, and pi-pi interactions [35]. The use of biochar, due to its chemical composition, can increase pH and Cation Exchange Capacity (CEC), and contain essential nutrients for plant growth, which can lead to further supportive effects [36]. These factors affecting soil properties could also influence the immobilisation of TMEs, especially pH.

The aim of this study was to investigate the application of plastic mulch and biochar separately and in combination at the planting stage for two *Miscanthus* hybrids planted by the rhizome method (TV1) and the seedling plug method (GNT43) on soils polluted with trace metals (Pb, Cd, Zn). The survival rate and morphological parameters were studied in the first and second growing seasons since planting, while the elemental composition was analyzed at the end of the first growing season. The research hypothesis was that the application of biochar and plastic mulch, both individually and in combination, will positively influence the establishment of *Miscanthus* hybrids on TME-contaminated soils. To our knowledge, this article is the first to present an approach for applying the above agrotechniques to improve the establishment of *Miscanthus* on TME-contaminated soils.

## 2. Results

**Figure 1:**
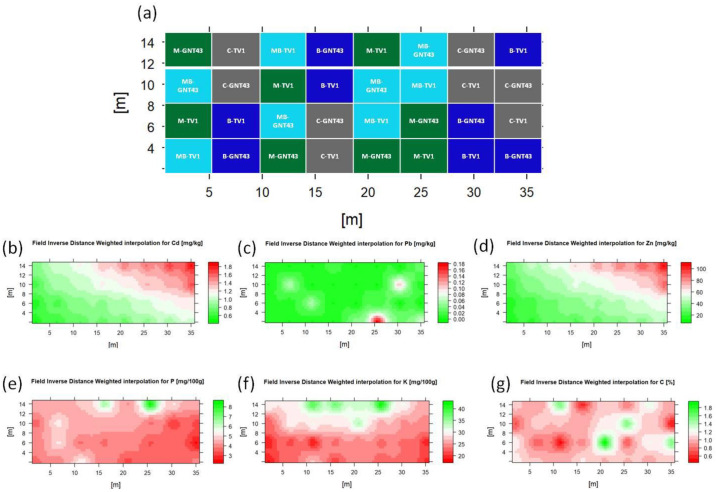
Trial design (**a**) and visualization of the bio-availability of selected elements in the soil (**b**–**g**). Color graduation for Pb, Cd, and Zn is from green to red, while for other visualised parameters, from red to green.

Under in situ conditions, in contrast to pot experiments, there is always surface runoff and the soil is not homogeneous. Therefore, it is important to randomise the plots, as shown in Figure 1a. Due to this randomisation, no significant differences in cropping conditions were found between the blocks, despite the rather clear gradient of TME contamination and assimilable nutrient distribution (Figure 1). Other parameters characterising the physicochemical properties of the soil did not differ statistically significantly between the experimental treatments (Table 1).

**Figure 2:**
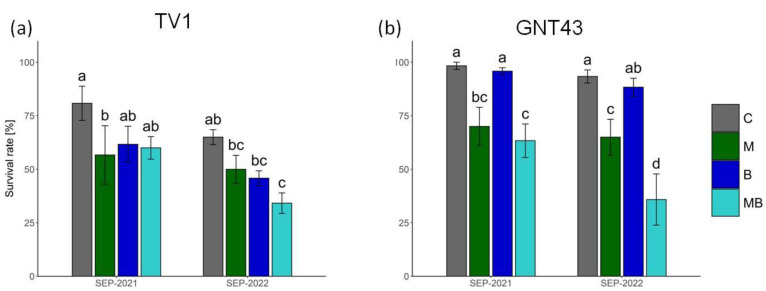
Survival rate of TV1 (**a**) and GNT43 (**b**) hybrids at the end of two successive growing seasons under different agrotechnical treatments: control (C), plastic mulch (M), biochar (B), and plastic mulch + biochar (MB). Presented values are means ± SD (*n* = 4). Means followed by the same letter in a chart are not significantly different from each other using the LSD test (*p* ≤ 0.05).

Both treatments had a significantly negative effect on the survival of the two *Miscanthus* hybrids tested (Figure 2). The TV1 hybrid reacted negatively to all treatments applied and the lowest survival rate was observed in plots with the combined plastic mulch and biochar (MB) (Figure 2a). For hybrid GNT43, the use of biochar (B) had no effect on plant survival compared to the control, whereas plastic mulch (M) and the combined treatment (MB) led to a significant decrease in survival compared to the control (Figure 2b).

**Figure 3:**
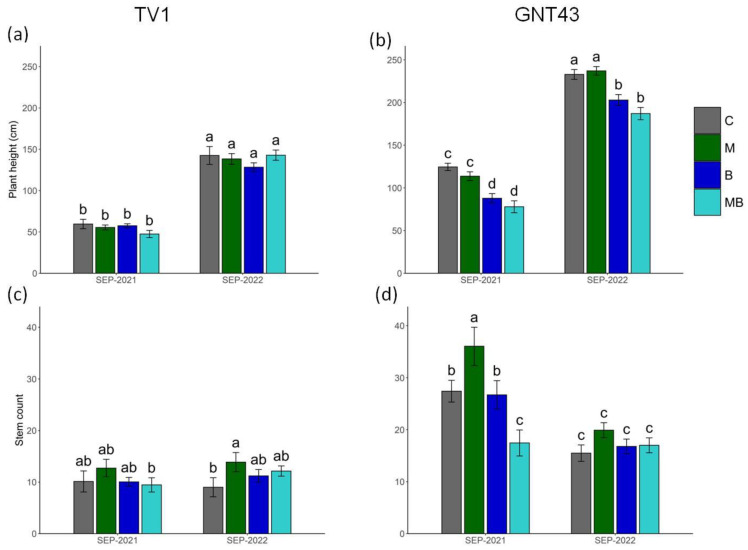
The impact of selected agrotechnical treatments on number of stems or plant height of TV1 (**a** and **c**, respectively) and GNT43 (**b** and **d**, respectively). Tested treatments: control (C), plastic mulch (M), biochar (B), and plastic mulch + biochar (MB). Presented values are means ± SD (*n* = 24; 6 measurements per plot, 4 plots per variant). Means followed by the same letter in a chart are not significantly different from each other using the LSD test (*p* ≤ 0.05).

For TV1, no significant effect of any treatments was observed on plant height, compared to the control (Figure 3a). However, B and the MB resulted in reduced plant height, compared to the C- and M-only treatments for GNT43 (Figure 3b).

Covering the seedlings with M at planting had a positive (though not always significant) effect on the stem number in both *Miscanthus* hybrids (Figure 3c,d). The use of B and MB had no significant effect on the number of stems, regardless of the plant hybrid.

There were no significant changes in the content of elements in the shoots of TV1 as a result of the agrotechnical treatments applied (Table 2); however, GNT43 was characterised by a significantly higher accumulation of metals in the shoots compared to the control when grown in the combined variant (MB) (Table 2).

**Figure 4:**
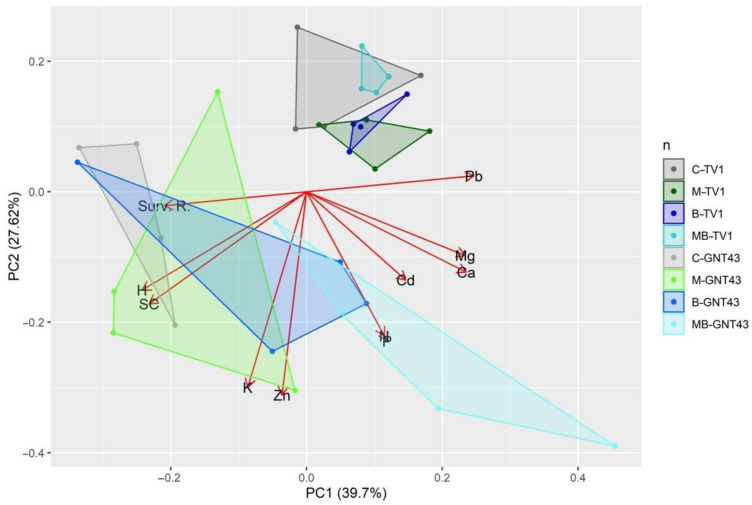
The Principal Component Analysis (PCA) presenting the relationships between selected elements accumulation in shoots and growth parameters of TV1 and GNT43 *Miscanthus* hybrids cultivated under different agrotechnical treatments: control (C), plastic mulch (M), biochar (B), and plastic mulch + biochar (MB). Growth parameters: H—plant height; SC—stem count; Surv. R.—survival rate.

The principal component analysis confirmed to some extent the absence of a significant influence of the agrotechnical measures applied on the growth and development of the hybrid TV1 (Figure 4). All cases within the treatments are clustered with a small distance between each other, and the formed groups overlap. However, different analysis results were obtained for the GNT43 hybrid. The agrotechnical treatments applied had a significant negative influence on the growth of the plants of this hybrid and led to an increased accumulation of metals in the shoots. The PCA also shows that the variation in TV1 between treatments is lower compared to GNT43.

## 3. Discussion

Anthropogenic activities, especially from the long-standing smelting of Pb and Zn in the last century, are responsible for widespread soil contamination with TMEs such as Pb, Cd, and Zn [37]. The additional influence of regular physical processes in the soil, such as leaching or runoff, can make the uneven distribution of these elements even more pronounced [38]. For this reason, proper randomization of field trials is important, especially for growing crops on soils contaminated with trace metals. The metal fractions in soil which directly influence the accumulation of TMEs by plants are the ion exchange or bio-available fraction [39,40]. The same is true for the assimilable fraction of phosphorus and potassium. Spatial distribution maps show a clear gradient of the bio-available Cd and Zn fractions associated with the above processes. Interestingly, there were no such observations for the bio-available fraction of Pb, which seems to be related to the low mobility of Pb in soil [22] and the generally low concentration ranges. Considering these differences in the spatial distribution of elements, the treatment-based soil analysis showed that there were no significant differences between plots with different treatments for most of the elements and physicochemical parameters measured in the soil, except for Fe and K. However, the assimilable forms of K (K_2_O), which can affect plant growth and development [41], showed no significant changes between plot treatments.

Survival after planting at the end of the first growing season and after the second growing season was strongly hybrid-dependent. In general, TV1 had a lower survival rate regardless of treatment. Clifton-Brown and Lewandowski [11] reported for different *Miscanthus* species (*M.* × *giganteus*, *Miscanthus sinensis*, and *Miscanthus sachariflorus*) that winter losses (%) were highest in the Scandinavian countries where *M.* × *giganteus* reached almost a 100% loss. Interestingly, only one hybrid (Sin-H6) showed a desirable success rate in these countries. Xue et al. [16] found that, of the propagation types studied, the highest survival rate was achieved with the rhizome-based approach; opposite results were obtained in this work. This could be due to two factors. The first relates to the fact that Xue et al. [16] did not investigate a mixed approach associated with planting cuttings pre-cultivated with seeds in the greenhouse before planting them out in the field. The second factor could be related to the hybrid response, which seems to be more relevant considering the morphological data. Contrary to expectations, agrotechnical treatments led to a significant reduction in survival, which was most evident in plastic mulch treatments. These results are inconsistent with previous reports on plastic mulch films by O’Loughlin et al. [14] and Ashman et al. [21]. On the one hand, this contrasting observation could be related to late planting, which could lead to high temperatures under the film, which could then trigger heat stress. On the other hand, the application of film could trigger undesirable crossing effects with TMEs in the soil.

The application of biochar without plastic mulch did not cause a significant decrease in survival compared to the control, but the tendency towards a decrease is visible in TV1. The worst results for survival were found for the combined treatment with plastic mulch and biochar. It is noteworthy that for this treatment, a further decrease in survival rate was found after the first winter, indicating the poor condition of the seedlings in the first growing season. Most literature data reporting studies of crop cultivation with biochar on soils contaminated with TMEs indicate that biochar reduces the mobility of TMEs in the soil, thus increasing the growth parameters and reducing the aboveground accumulation of TMEs [42,43,44]. The separate and combined application of biochar did not show significant differences in the number of TV1 stems and plant height between these treatments compared to the control, in the first and the second growing season. However, there were differences in the GNT43 plants, especially in plant height, suggesting that biochar treatment alone and in combination led to a reduction in plant height. At this stage, the accumulation of TMEs in the aboveground plants is important. For TV1, there were no significant differences in aboveground TME concentration or macro- and micronutrient concentration regardless of treatment, but significant differences were found for GNT43.

All treatments for GNT43 were characterised by significantly higher Pb and Cd concentrations in the aboveground parts. This observation is particularly unexpected for biochar treatments and surpasses most reports on this topic. Zhang et al. [45] observed similar behavior to this report. The authors found that lettuce cultivated in lightly and heavily Cd-contaminated soils showed different growth and Cd accumulation behavior. Lettuce grown in heavily contaminated soils with biochar amendment showed a higher Cd concentration compared to the control. It was discussed that this behavior might be related to the increase in lettuce root biomass caused by the improved physical and biological properties of the soil after biochar application, which in turn led to further uptake and transport of Cd from the soil into the shoot. This observation seems true considering that biochar application in this experiment improved the soil conditions for the seedlings right at the beginning of the growth stages by increasing the absorption surface and thus increasing the uptake at later stages for GNT43. It is worth noting that the total Cd concentration in this experiment was about 3–4 times higher compared to the report by Zhang et al. [45]. The other important factor that could be related to the fact that there is no difference in the concentration of the elements in the aboveground parts of the rhizome-planted TV1 could be related to the longer time required for the development of the root system compared to the already developed root system of the plug-planted GNT43. This fact could also influence the survival rate. Further research is needed to confirm this hypothesis, as this would require an in-depth study of root morphology at different times after planting. Considering that biodegradable plastic mulch is the major contributor to the temperature increase at the beginning of planting [14]. Rajkumar et al. [46] found that soil warming can affect the nutrient and TME pathway by altering the release of soluble metal ions into the soil solution through the decomposition of SOM, analysis of microbial cells, and destruction of soil aggregates, thereby changing the bio-availability of metals, their uptake, and distribution in plant tissues. This statement is consistent with the results obtained for the GNT43 treatments with plastic mulch, especially for Pb and Cd.

Mature field plantations (>3 years) of *Miscanthus* hybrids on TME-contaminated soils do not accumulate TMEs [22,23,47]. However, this observation does not hold for the first growing season as the concentration is much higher [24,25,26], which could be associated with reduced survival and growth, as confirmed in this study. For this reason, the calculation of phytoremediation indicators such as the bio-accumulation factor or the translocation factor [48] could give false positive results for phytoextraction potential in the first growing season. Contrary to expectations, both treatments caused a decrease in survival and an increase in TMEs in biomass during the first growing season, which is critical for the further success of the planting. Further studies are needed to investigate the method (i.e., mineral amendments, microbial inoculants, different coating materials) that could truly reduce the impact of TMEs on plant growth and development and thus increase survival rates. The same applies to the planting method and variety selection.

## 4. Materials and Methods

### 4.1. Trial Design

The field trial was established on arable land contaminated with lead, cadmium, and zinc (50°20′41.814′′ N 18°57′20.224′′ E) from historical smelting activity nearby. In recent years, mainly cereals have been cultivated there. Before planting, soil was ploughed and harrowed to produce a fine tilth. The trial design was a split plot block including four replicates. Each plot (15 m^2^) contained 30 plants. The trial design included two *Miscanthus* hybrids, rhizome-based TV1 and seed-based GNT43, with four treatments: control (C), biochar (B) (Supplementary Materials Table S1), biodegradable plastic mulch (M), and combined biochar and plastic mulch (BM). Wood-based biochar (Table S1) was added to the planting hole as 5% *v*/*v* and thoroughly mixed with the soil. Plastic mulch was applied immediately after planting. All planting was done manually in mid-June 2021. Plant material was provided by Terravesta Ltd. (TV1 hybrid, Lincoln, UK) and Hohenheim University (GNT43 hybrid, Stuttgart, Germany). No fertilization or plant protection products were applied on the field. Manual weeding was done regularly during the first growing season. Plant survival was assessed at the end of the first growing season in September 2021 and at the start of the second growing season in May 2022, expressed as a percentage of plant survival per plot. Plant measurements (stem number and height) were assessed in September 2021 and 2022, while plant samples for elemental analyses were taken in March 2022. Weather data regarding temperature and precipitation were collected from a meteorological station located about 10 km from the trial.

### 4.2. Soil and Plants Properties

Soil samples were collected from each plot before planting. Soil samples from different segments of each plot were used for analysis and served as the basis for the creation of further spatial maps, with the sampling points for analysis located in the centre of each plot. Soil physicochemical parameters were measured on the material sieved through a 2 mm sieve. The pH of the soil was measured in H_2_O (ratio 1:2.5 *m*/*v*) and KCl using a combined glass/calomel electrode (OSH 10-10, METRON, Gliwice, Poland) and a pH meter (CPC-551, Elmetron, Gliwice, Poland) at 20 °C. Electrical conductivity (EC) was determined with an ESP 2ZM electrode (EUROSENSOR, Gliwice, Poland) according to the Polish standard [49]. Soil texture was assessed using the hydrometric method according to the Polish standard [50]. Soil organic matter (SOM) content was measured by loss on ignition as follows: 5 g of air-dry soil was dried at 105 °C for 24 h and then treated at 550 °C for four hours. Soil organic carbon (C_org_) was assessed according to [51]. The total concentrations of metals in soil (<0.25 mm) and in plants were analysed using a flame atomic absorption spectrometer (iCE 3500 FAAS, Thermo Scientific, Waltham, MA, USA) after microwave sample digestion (ETHOS 1, Milestone, Sorisole, Italy) according to the procedure specified by the manufacturer (concentrated HNO_3_ and H_2_O_2_, 4:1 *v*/*v*). Bio-available Cd, Pb, and Zn was analyzed after extraction with 0.01 mol/dm^3^ CaCl_2_, following methods described in Pogrzeba et al. [39], and measured using a flame atomic absorption spectrometer (SpektrAA 300, Varian INC., Palo Alto, CA, USA). The concentrations of available phosphorus and potassium were determined according to the method described by Egnér et al. [52].

### 4.3. Statistical Analysis

The results are shown as means ± SE. The statistical significance of the differences was determined using one-way ANOVA and the post hoc Fisher’s LSD test (*p* < 0.05). PCA was used to identify the dominant groups of the factors that determine and describe the variety differences. Relative coordinates spatial distribution maps of assimilable elements were constructed based on plot elemental analysis data and Inverse Distance Weighted (IDW) interpolation. The software used for all statistical analyses was R (version 4.2.0., R Foundation for Statistical Computing, Vienna, Austria).

## 5. Conclusions

The survival rate of the *Miscanthus* hybrids studied was highly hybrid-dependent and, additionally, influenced by the particular planting technique. The TV1 planted with rhizomes had a lower survival rate (about 60%) and was characterised by weaker growth (height and number of stems) compared to the seed-based GNT43, where the survival rate was over 90% in the most effective treatment, which was the control for both hybrids. The agrotechniques applied in this experiment to improve the establishment rate had an opposite effect, and of all the treatments, the greatest plant losses occurred in the combined biochar and plastic mulch treatment, regardless of hybrid. TV1 showed no statistical difference in element concentration between treatments after the first growing season, while significantly higher concentrations of Pb and Cd were found in GNT43, compared to the control, regardless of treatment. Contrary to published literature with uncontaminated soils, the separate or joint application of biochar and plastic mulch neither increased the survival rate nor reduced the accumulation of toxic TMEs during establishment on contaminated soils. Despite this, *Miscanthus*, especially GNT43, once established, grows very well with low offtakes, and in the absence of alternatives, these marginal contaminated areas should be expanded with *Miscanthus* hybrids. There is a need for further research with different approaches to help the establishment of *Miscanthus* hybrids in the first year after planting on difficult soils, i.e., contaminated with TMEs. Agronomic developments for various marginal contaminated soils in different countries need to be further developed commercially.

## Figures and Tables

**Table 1:** The soil physicochemical characteristics on experimental plots.

Variety	TV1				GNT43			
Treatment	C	M	B	MB	C	M	B	MB
Physicochemical Parameters								
pH_H2O_	7.11 ± 0.06 a	7.15 ± 0.04 a	7.06 ± 0.04 a	7.14 ± 0.04 a	7.07 ± 0.06 a	7.17 ± 0.06 a	7.07 ± 0.06 a	7.15 ± 0.06 a
pH_KCl_	6.39 ± 0.14 a	6.50 ± 0.11 a	6.38 ± 0.09 a	6.49 ± 0.08 a	6.42 ± 0.06 a	6.41 ± 0.15 a	6.40 ± 0.11 a	6.48 ± 0.06 a
EC [µS cm^−1^]	75.7 ± 8.4 a	74.8 ± 0.7 a	74.4 ± 2.8 a	76.9 ± 7.4 a	67.2 ± 2.7 a	79.4 ± 7.1 a	73.5 ± 6.8 a	72.4 ± 2.8 a
Pb_bio_ [mg kg^−1^]	0.007 ± 0.005 a	0.024 ± 0.014 a	0.032 ± 0.018 a	0.003 ± 0.003 a	0.011 ± 0.008 a	0.001 ± 0.001 a	0.033 ± 0.025 a	0.045 ± 0.042 a
Cd_bio_ [mg kg^−1^]	1.18 ± 0.22 a	0.95 ± 0.20 a	1.12 ± 0.19 a	0.94 ± 0.11 a	0.98 ± 0.12 a	1.13 ± 0.22 a	1.25 ± 0.23 a	0.82 ± 0.10 a
Zn_bio_ [mg kg^−1^]	55 ± 14 a	41 ± 14 a	51 ± 13 a	38 ± 6 a	41 ± 8 a	52 ± 13 a	60 ± 16 a	32 ± 6 a
OM [%]	5.75 ± 0.12 ab	5.75 ± 0.15 ab	5.98 ± 0.15 ab	6.08 ± 0.16 a	5.83 ± 0.14 ab	5.90 ± 0.24 ab	6.00 ± 0.15 ab	5.60 ± 0.16 b
P_2_O_5_ [mg 100g^−1^]	4.34 ± 0.72 a	4.73 ± 1.19 a	4.46 ± 0.38 a	4.38 ± 0.41 a	3.90 ± 0.54 a	4.05 ± 0.23 a	3.97 ± 0.33 a	3.97 ± 0.19 a
K_2_O [mg 100g^−1^]	26.6 ± 3.4 a	26.8 ± 5.1 a	25.8 ± 2.6 a	28.4 ± 2.3 a	23.1 ± 2.0 a	29.1 ± 4.3 a	25.4 ± 1.8 a	23.7 ± 2.4 a
C_org_ [%]	0.85 ± 0.16 b	0.77 ± 0.09 b	1.06 ± 0.08 ab	0.98 ± 0.09 ab	1.04 ± 0.17 ab	1.34 ± 0.13 a	1.00 ± 0.04 ab	1.27 ± 0.20 a
Pb [mg kg^−1^]	450 ± 23 a	442 ± 29 a	460 ± 22 a	463 ± 14 a	438 ± 21 a	454 ± 35 a	483 ± 17 a	423 ± 25 a
Cd [mg kg^−1^]	17.9 ± 0.7 a	17.8 ± 1.2 a	18.4 ± 0.7 a	18.6 ± 0.4 a	17.3 ± 0.8 a	18.1 ± 1.3 a	19.2 ± 0.4 a	16.8 ± 1.1 a
Zn [mg kg^−1^]	1910 ± 60 a	1880 ± 110 a	1920 ± 70 a	2000 ± 30 a	1830 ± 70 a	1910 ± 130 a	2030 ± 20 a	1800 ± 120 a
Mg [mg kg^−1^]	2400 ± 390 a	2410 ± 150 a	2290 ± 170 a	2750 ± 310 a	2260 ± 110 a	2410 ± 230 a	2410 ± 270 a	2410 ± 90 a
P [mg kg^−1^]	767 ± 44 a	790 ± 30 a	801 ± 19 a	811 ± 16 a	740 ± 23 a	774 ± 46 a	811 ± 33 a	769 ± 34 a
K [mg kg^−1^]	1120 ± 50 ab	1120 ± 20 ab	1120 ± 30 ab	1190 ± 40 a	1060 ± 30 b	1140 ± 60 ab	1130 ± 40 ab	1150 ± 30 ab
Ca [mg kg^−1^]	3960 ± 730 a	4050 ± 280 a	3800 ± 320 a	4720 ± 570 a	3690 ± 210 a	4000 ± 360 a	4020 ± 500 a	3930 ± 120 a
Fe [mg kg^−1^]	15,500 ± 250 b	16,200 ± 350 ab	15,800 ± 150 ab	16,500 ± 350 ab	16,100 ± 350 ab	16,000 ± 400 ab	16,600 ± 60 a	16,000 ± 500 ab

Presented values are means ± SD (*n* = 4). Means followed by the same letter in a row are not significantly different from each other using the LSD test (*p* ≤ 0.05). Abbreviations: C—control; M—plastic mulch; B—biochar treatment; MB—combined treatment with plastic mulch and biochar; EC—electrical conductivity of soil solution; OM—organic matter; C_org_—organic carbon; Pb_bio_, Cd_bio_, Zn_bio_—Ion-exchangable fraction of metals.

**Table 2:** Accumulation of selected elements in shoots of TV1 or GNT43 hybrids under different agrotechnical treatments.

Variety	TV1				GNT43			
Treatment	C	M	B	MB	C	M	B	MB
Pb [mg kg^−1^]	30.8 ± 6.3 ab	34.1 ± 4.6 a	29.6 ± 1.5 ab	33.3 ± 2.7 a	16.5 ± 0.5 c	19.5 ± 2.8 bc	29.4 ± 6.3 ab	31.0 ± 5.7 ab
Cd [mg kg^−1^]	1.02 ± 0.07 b	1.05 ± 0.17 b	1.00 ± 0.07 b	0.98 ± 0.12 b	0.80 ± 0.03 b	1.16 ± 0.23 ab	1.17 ± 0.17 ab	1.51 ± 0.14 a
Zn [mg kg^−1^]	170 ± 20 b	165 ± 17 b	166 ± 12 b	161 ± 13 b	248 ± 16 a	299 ± 28 a	309 ± 35 a	293 ± 48 a
Ca [mg kg^−1^]	1510 ± 140 b	1570 ± 160 ab	1520 ± 130 b	1490 ± 110 b	1230 ± 50 b	1240 ± 150 b	1510 ± 250 b	2060 ± 290 a
Mg [mg kg^−1^]	890 ± 50 abc	910 ± 50 abc	950 ± 110 ab	880 ± 20 bc	680 ± 50 c	760 ± 50 bc	800 ± 110 bc	1110 ± 130 a
N [%]	0.93 ± 0.07 ab	1.03 ± 0.03 ab	1.01 ± 0.07 ab	0.95 ± 0.03 ab	0.87 ± 0.06 b	1.00 ± 0.14 ab	1.18 ± 0.16 a	1.17 ± 0.10 a
P [mg kg^−1^]	950 ± 80 b	1210 ± 30 ab	1190 ± 90 ab	970 ± 90 b	1090 ± 180 ab	1020 ± 190 ab	1130 ± 150 ab	1390 ± 150 a
K [mg kg^−1^]	6430 ± 600 d	7970 ± 530 cd	7220 ± 570 cd	6440 ± 650 d	14,880 ± 1940 ab	16,000 ± 3390 ab	11,680 ± 1010 bc	17,650 ± 2760 a

Presented values are means ± SD (*n* = 4). Means followed by the same letter in a row are not significantly different from each other using the LSD test (*p* ≤ 0.05). C—control, M—plastic mulch, B—biochar, MB—plastic mulch + biochar.

## Data Availability

Not applicable.

## Supplementary Materials

The following supporting information can be downloaded at: https://www.mdpi.com/article/10.3390/plants12010098/s1; Table S1: Biochar properties, TerrAfix Ltd. UK; Figure S1: Meteorological data for experiment period (2021–2022) for Bytom site.
