# Supplementary material for: The Effect of Different Agrotechnical Treatments on the Establishment of Miscanthus Hybrids in Soil Contaminated with Trace Metals
Source: Plants (Basel). 2022 Dec 25;12(1):98. doi: 10.3390/plants12010098 (PMC9823936; doi:10.3390/plants12010098)

Table S1. Biochar properties, TerrAfix Ltd. UK (n=5), bdl – below detection limit

Biochar	
**Parameter**	
pH (H2O)	9,4 ±0,08
pH (KCl)	8,3 ±0,13
EC [μS/cm]	110,1 ±12
Pb [mg/kg]	2,2 ±0,23
Cd [mg/kg]	bdl
Zn [mg/kg]	47,5 ±2,34
As [mg/kg]	0,3 ±0,08
P [mg/kg]	337 ±7
K [mg/kg]	2738 ±165
Mg [mg/kg]	804 ±15
Cl [%]	0,1 ±004
C [%]	77,8 ±0,86
H [%]	3,1 ±0,05
N [%]	0,3 ±0,01
S [%]	0,01 ±0,002

Figure S1. Meteorological data for experiment period (2021-2022) for Bytom site

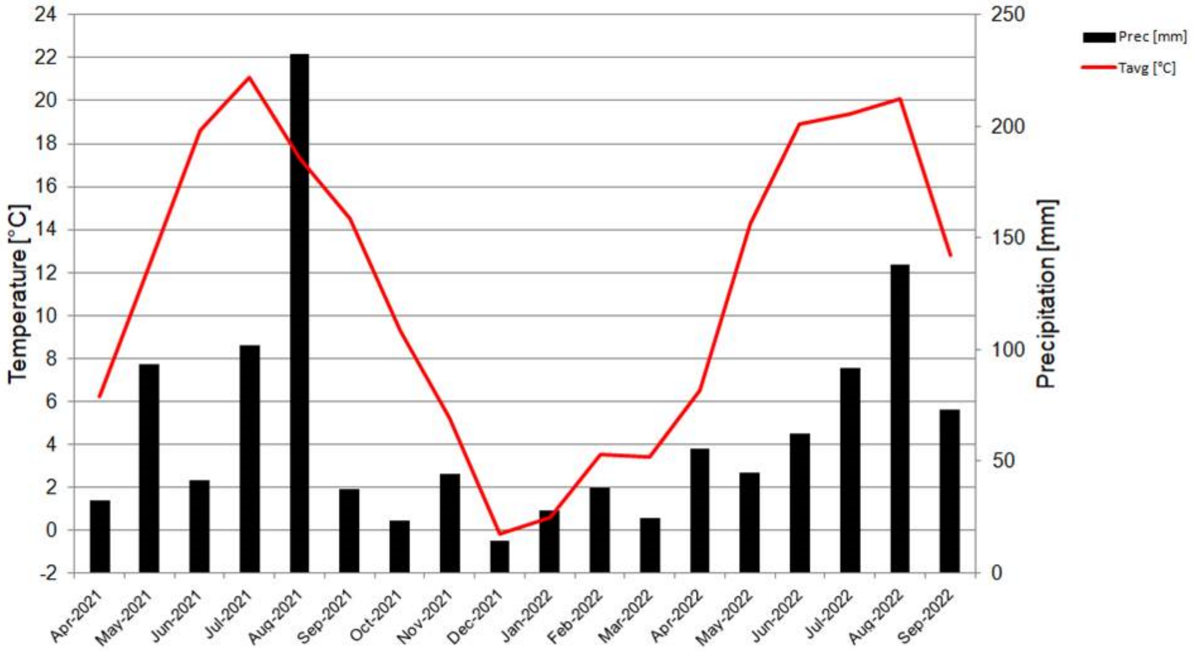

Supplement: Supplementary file 1 [file plants-12-00098-s001.zip › plants-2125145-supplementary.pdf]
